# Upregulation of Phosphodiesterase 2A Augments T Cell Activation by Changing cGMP/cAMP Cross-Talk

**DOI:** 10.3389/fphar.2021.748798

**Published:** 2021-10-05

**Authors:** Roberta Kurelic, Paula F. Krieg, Jana K. Sonner, Gloria Bhaiyan, Gustavo C. Ramos, Stefan Frantz, Manuel A. Friese, Viacheslav O. Nikolaev

**Affiliations:** ^1^ Institute of Experimental Cardiovascular Research, University Medical Center Hamburg-Eppendorf, Hamburg, Germany; ^2^ Institute of Neuroimmunology and Multiple Sclerosis, Center for Molecular Neurobiology Hamburg, University Medical Center Hamburg-Eppendorf, Hamburg, Germany; ^3^ Department of Internal Medicine I, University Hospital Würzburg, Würzburg, Germany; ^4^ Comprehensive Heart Failure Centre, University Hospital Würzburg, Würzburg, Germany; ^5^ German Center for Cardiovascular Research (DZHK), Partner Site Hamburg/Kiel/Lübeck, Hamburg, Germany

**Keywords:** cAMP, T cells, phosphodiesterase, cGMP/cAMP cross-talk, FRET

## Abstract

3′,5′-cyclic adenosine monophosphate (cAMP) is well-known for its diverse immunomodulatory properties, primarily inhibitory effects during T cell activation, proliferation, and production of pro-inflammatory cytokines. A decrease in cAMP levels, due to the hydrolyzing activity of phosphodiesterases (PDE), is favoring inflammatory responses. This can be prevented by selective PDE inhibitors, which makes PDEs important therapeutic targets for autoimmune disorders. In this study, we investigated the specific roles of PDE2A and PDE3B in the regulation of intracellular cAMP levels in different mouse T cell subsets. Unexpectedly, T cell receptor (TCR) activation led to a selective upregulation of PDE2A at the protein level in conventional T cells (Tcon), whereas no changes were detected in regulatory T cells (Treg). In contrast, protein expression of PDE3B was significantly higher in both non-activated and activated Tcon subsets as compared to Treg, with no changes upon TCR engagement. Live-cell imaging of T cells expressing a highly sensitive Förster resonance energy transfer (FRET)-based biosensor, Epac1-camps, has enabled cAMP measurements in real time and revealed stronger responses to the PDE2A inhibitors in activated vs non-activated Tcon. Importantly, stimulation of intracellular cGMP levels with natriuretic peptides led to an increase of cAMP in non-activated and a decrease of cAMP in activated Tcon, suggesting that TCR activation changes the PDE3B-dependent positive to PDE2A-dependent negative cGMP/cAMP cross-talk. Functionally, this switch induced higher expression of early activation markers CD25 and CD69. This constitutes a potentially interesting feed-forward mechanism during autoimmune and inflammatory responses that may be exploited therapeutically.

## Introduction

3′,5′-cyclic adenosine monophosphate (cAMP) is a ubiquitous second messenger with prominent immunomodulatory functions (Mosenden and Taskén, 2011). Increased cAMP formation in regulatory T cells (Treg) is crucial for their capacity to suppress conventional T cells (Tcon) (Bopp et al., 2007). Mechanistically, Treg immunosuppression is mediated through several complementary mechanisms, including a direct transfer of cAMP from Treg to Tcon through gap junctions, and the degradation of extracellular adenosine triphosphate (ATP) to adenosine via ectoenzymes present on the surface of Treg (CD39 and CD73) and subsequent engagement of adenosine receptors on Tcon (Klein and Bopp, 2016). Intracellular cAMP levels are balanced by the activity of adenylyl cyclases (AC) and cyclic nucleotide phosphodiesterases (PDE) (Brescia and Zaccolo, 2016). Stimulation of β-adrenergic receptors (β-AR) and adenosine receptors on the surface of T cells activates ACs which catalyze the formation of cAMP, while PDEs control cyclic nucleotide homeostasis by hydrolyzing cAMP to 5ʹ-AMP (Conti and Jin, 1999; Borea et al., 2018). PDEs are comprised of 11 enzyme families (PDE1-11) containing more than 100 isoforms (Conti and Beavo, 2007). Some of them have already well-established roles in T cell physiology, such as PDE3, PDE4, PDE7, and PDE8 (Giembycz et al., 1996; Glavas et al., 2001; Abrahamsen et al., 2004; Ruppelt et al., 2007; Basole et al., 2017; Epstein et al., 2021). PDE3B, PDE4B, and PDE8A have been evaluated by immunoblotting and qPCR and showed higher expression in naїve Tcon as compared to Treg (Vang et al., 2013). Also, it has previously been described that forkhead box transcription factor (*Foxp3*), a transcriptional master regulator of Treg, inhibits *Pde3b* gene transcription, thereby maintaining higher cAMP levels in these cells (Gavin et al., 2007; Huang et al., 2009). Also, PDE2 and PDE4 together were identified as a major contributors to total PDE activity in murine thymocytes (Michie et al., 1996). Yet, the role of PDE2A in the regulation of cyclic nucleotide pools and function in T cells remains unclear.

PDE2, with its unique subfamily PDE2A, is a dual-substrate PDE known to hydrolyze both cyclic nucleotides, cAMP and 3′,5′-cyclic guanosine monophosphate (cGMP) (Martins et al., 1982). The hydrolytic activity of PDE2A can be stimulated by cGMP to reduce cAMP levels, hence its alternative name is cGMP-stimulated PDE. Binding of cGMP to the allosteric GAF-B domain of PDE2A initiates a negative cGMP/cAMP cross-talk and degradation of cAMP occurs at a higher rate (Bender and Beavo, 2006). cGMP generation in cells is catalyzed either by nitric oxide (NO) sensitive soluble (sGC) or by particulate guanylyl cyclase (pGC), which represents a family of membrane receptors for natriuretic peptides (NP). Atrial (ANP) and brain (BNP) natriuretic peptides bind to and activate guanylyl cyclase-A (GC-A; also called natriuretic peptide receptor-A, NPRA), while C-type natriuretic peptide (CNP) activates guanylyl cyclase-B (GC-B; also named natriuretic peptide receptor-B, NPRB) (Zaccolo and Movsesian, 2007; Weber et al., 2017). Along with PDE2A involvement in the interplay between cyclic nucleotides, PDE3 has a similar central role. PDE3 is also known as cGMP-inhibited PDE although it has a high affinity for both cAMP and cGMP (Degerman et al., 1997). However, cGMP binds predominantly to the catalytic domain of PDE3 and acts as a competitive inhibitor of cAMP hydrolysis resulting in a positive cGMP/cAMP cross-talk loop (Zaccolo and Movsesian, 2007). Thus, the accumulation of cGMP in the cytosol either facilitates cAMP hydrolysis through the rise in PDE2A activity or competitively inhibits PDE3 activity which culminates in a net increase of cAMP levels.

Exploring the dynamics of cyclic nucleotide changes at the single-cell level has gained greater interest with the development of fluorescent biosensors for live cell imaging. Based on the phenomenon known as Förster resonance energy transfer (FRET) and combined with fluorescent microscopy, they serve as essential tools in tracing alterations of intracellular cAMP and cGMP signaling with immense spatial and temporal resolution (Börner et al., 2011). A plethora of fluorescent biosensors have previously been used to directly visualize changes related to β-AR dependent cyclic nucleotide signaling in healthy and diseased cells, especially in cardiomyocytes (Mehel et al., 2013; Perera et al., 2015). More than a decade ago, real-time live-cell imaging of cAMP in multiple primary cell types became possible due to the generation of transgenic mice expressing a highly sensitive FRET–based biosensor, Epac1-camps, in an ubiquitous fashion (Calebiro et al., 2009). Transgenic sensor animals bring multiple benefits in performing live-cell microscopy, such as uniform sensor expression in the cell as well as the possibility to investigate cyclic nucleotide signaling pathways in intact, freshly isolated cells (Börner et al., 2011). However, up until now, real-time cAMP measurements have not been performed in T cells, especially not to entangle the PDE-mediated cGMP/cAMP cross-talk.

In the present study, we analyzed cGMP/cAMP cross-talk and its regulation in primary murine T cells. We found that PDE2A was selectively upregulated upon T cell receptor (TCR) engagement in Tcon, which led to a turn-around of the cGMP/cAMP cross-talk and facilitated higher expression of early activation markers CD25 and CD69.

## Methods

### Animals

Eight to twelve-week-old CAG-Epac1-camps and DEREG transgenic mice, as well as C57BL/6 wildtype mice, were used in this study. Generation of the CAG-Epac1-camps transgenic mice on FVB/N background was previously described (Calebiro et al., 2009)**.** Briefly, in this transgenic mouse model, the cAMP sensor, Epac1-camps, is ubiquitously expressed under the control of cytomegalovirus enhancer/chicken β-actin (CAG) promoter in almost all tissues and cells (except erythrocytes and hair). Mice were kept under specific pathogen-free conditions in the central animal facility of the University Medical Center Hamburg-Eppendorf (UKE) and were handled according to international and national animal welfare guidelines with the organ isolation procedures approved by the *Behörde für Justiz und Verbraucherschutz* Hamburg (ORG1010, ORG946). Protocol for inducing experimental autoimmune encephalomyelitis and tissue sample collection was approved by the same authority (Nr 45/17).

### Chemicals

Mouse (atrial NP, 1–28) and human natriuretic peptides (C-type NP, 1–53) were purchased from Bachem. BAY 60–7,550 (BAY) was ordered from Santa Cruz. 3-isobutyl-1-methylxanthine (IBMX) was obtained from AppliChem. All other chemicals were from Sigma-Aldrich.

### T Cell Isolation and Activation

Lymph nodes (superficial cervical, axillary, brachial, inguinal) and spleen were separately collected in ice-cold PBS. For immunoblotting single-cell suspensions were processed using the murine CD4^+^CD25^+^ Regulatory T cell Isolation Kit (Miltenyi Biotec) according to manufacturer’s instructions to obtain Treg and Tcon T cell subsets. For FRET measurements single-cell suspensions were processed using the murine CD4^+^ T cell Isolation Kit (Miltenyi Biotec) to enrich for CD4^+^ T cells via negative selection based on the manufacturer’s protocol and further labeled with CD25-Biotin antibody (Miltenyi Biotec) to separate CD4^+^CD25^+^ T cells from CD4^+^CD25^−^ T cells. T cell subsets were cultured 16–24 h in the presence or absence of Dynabeads Mouse T-Cell Activator CD3/CD28 (Thermo Fisher).

### Immunoblot Analysis

Murine T cells were centrifuged at 400x g for 4 min at 4°C, washed twice with ice-cold PBS, and lysed in RIPA buffer supplemented with Halt protease inhibitor cocktail (100x, Thermo Scientific). An equal number of cells was used continuously throughout the experiment. Samples were boiled at 95°C for 5 min. Protein lysates were loaded on 10% gel and SDS-PAGE was run followed by protein transfer onto nitrocellulose membrane (Amersham). Membranes were blocked in 3% or 5% non-fat milk prepared in TBS-T buffer, depending on the antibody, for 1 h at room temperature (RT). Subsequently, indicated primary antibodies were used in overnight incubation at 4°C: anti-PDE2A (1:750, Fabgennix), anti-PDE3B (1:2000, kindly provided by Dr. Sergei Rybalkin), anti-PDE4B (1:2500, Abcam) and anti-PDE4D (1:2500, Abcam). Anti-GAPDH (1:160000, Bio Trend) was used as a loading control in 30 min incubation at RT. After probing with the primary antibody, membranes were washed with TBS-T buffer three times and incubated for 1 h at RT with horseradish peroxidase (HRP)-conjugated secondary antibody (1:5000, goat anti-mouse or goat anti-rabbit, Biorad). To detect the signal, the SuperSignal West Pico PLUS Kit (Thermo Scientific) was used according to the manufacturer’s protocol. Densitometry analyses were performed on scanned blots in ImageJ software.

### FRET Measurements and Live Cell Imaging

T cell subsets were obtained as previously described. T cells were plated to glass coverslips coated with poly-D-lysine (Sigma-Aldrich) for approximately 30–45 min before live cell imaging was performed. Coverslips with plated T cells were placed in the Autofluor cell chamber, non-adherent cells were removed with FRET buffer (144 mM NaCl, 5.4 mM KCl, 1 mM MgCl_2_, 1 mM CaCl_2_, 10 mM HEPES; pH = 7.3) and 400 µl of FRET buffer was subsequently added into the chamber. Data were obtained by monitoring FRET response with an inverted fluorescent microscope, Leica DMI 3000 B, equipped with an oil-immersion 63x/1.40 objective and a MicroManager 1.4. software. CoolLED, as a single wave emitting diode, was used as the fluorescent light source to excite cyan fluorescent protein (CFP) at 440 nm. Beam-splitter, DV2 Dual View (Photometrics), was used to split the emission light into CFP and yellow fluorescent protein (YFP) channel, simultaneously monitored on a CMOS (OptiMOS, QImaging) camera chip. Images were produced every 10 s. After reaching the stable baseline, different chemical compounds, diluted in FRET buffer, were added to the chamber in a volume of 400 µl. Raw data obtained by the aforementioned fluorescent microscope were analyzed in ImageJ and corrected offline with a spectral bleedthrough correction factor using Microsoft Excel.

### T Cell Activation Assay and Flow Cytometry Analysis

For T cell activation assay, lymph nodes (superficial cervical, axillary, brachial, inguinal) and spleen from C57BL/6 wildtype mice were collected in ice-cold PBS. CD4^+^ T cells were isolated from single-cell suspensions using MojoSort CD4^+^ T cell isolation kit (BioLegend) according to manufacturer’s protocol. CD4^+^ T cells were seeded in an anti-CD3 (clone 145-2C11, BioLegend)-coated 96-well U-shape plate. Cells were supplemented with compounds (ANP and BAY) and soluble anti-CD28 (clone 37.51, BioLegend). Samples were incubated at 37°C and 5% CO_2_ for 6 h. After incubation, cells were stained for T cell surface marker for 30 min at 4°C. After fixation and permeabilization with eBioscience™ Foxp3/Transcription Factor Staining Buffer Set (Thermo Fisher), Foxp3 was stained for 30 min at RT. The following antibodies were used: CD3ε (clone 145-2C11, BioLegend), CD4 (clone GK1.5, BioLegend), CD8a (clone 53–6.7, BioLegend), CD69 (clone H1.2F3, BioLegend), CD25 (clone PC61, BioLegend) and Foxp3 (clone FJK-16s, Thermo Fisher). Dead cells were excluded by adding Alexa Fluor 750 NHS Ester (Succinimidyl Ester) (Thermo Fisher). Nonspecific Fc receptor-mediated antibody binding was minimized by blocking with TruStain FcX anti-mouse CD16/32 (clone 93, BioLegend). Samples were acquired on the BD FACS LSR II analyzer (BD Bioscience) or FACSymphony A3 (BD Bioscience). Data were analyzed using FlowJo (version 10, BD Bioscience) (Supplementary Figure S1). ANP-mediated changes were indicated by calculating an activation index, denoted as the difference of CD25 or CD69 expression between compound-treated unstimulated and compound-treated anti-CD3/CD28 stimulated cells.

### Experimental Mouse Model of Autoimmune Encephalomyelitis and Quantitative Real-Time PCR Analysis

For induction of experimental autoimmune encephalomyelitis (EAE) 8–9 weeks old female B6-DEREG (C.B6-Tg(Foxp3-DTR/EGFP)23.2Spar/Mmjax) mice were immunized subcutaneously with 100 μg MOG_35-55_ (peptides and elephants) in complete Freund’s adjuvant (BD Difco) containing 4 mg/ml *Mycobacterium tuberculosis* (BD Difco). 250 ng pertussis toxin (Merck Millipore) were injected intraperitoneally in 100 μl PBS on the day of immunization and 48 h later. Animals were scored daily from d7 for clinical signs of disease. At the peak of disease (d14/d15), immunized mice were sacrificed. Age and gender-matched naïve mice were sacrificed as healthy controls. T cells from acute EAE mice and healthy controls were isolated from the peripheral lymphatic organs (axillary, brachial, superficial cervical, inguinal lymph nodes and spleen). T cells were isolated using the MojoSort CD4^+^ T cell isolation kit (BioLegend) according to manufacturer’s instructions. Immune cells were identified by staining with following antibodies: CD4 (clone 145-2C11), TCRb (clone RM4-5), CD8a (clone 53–6.7), CD19 (clone 6D5) and CD11b (clone M1/70) (all BioLegend). Dead cells were excluded by adding Alexa Fluor 750 NHS Ester (Succinimidyl Ester) (Thermo Fisher). Nonspecific Fc receptor-mediated antibody binding was minimized by blocking with TruStain FcX anti-mouse CD16/32 (clone 93, BioLegend). Tcon and Treg were sorted with the FACS Aria III cell sorter (BD Bioscience). RNA was isolated using RNeasy Mini Kit (Qiagen) and cDNA was synthesized with RevertAid H Minus First Strand cDNA Synthesis Kit (Thermo Fisher). For quantitative real-time PCR, following TaqMan Gene Expression Assays were used: Mm01277042_m1 (mouse Tbp) and Mm01136644_m1 (mouse Pde2a) (both Thermo Fisher). Samples were analyzed with QuantStudio 6 Flex Real-Time PCR System (Thermo Fisher) and by using the ΔCt method for relative quantification.

### Statistics

Data shown in bar graphs are presented as mean ± standard error of the mean (SEM). In immunoblot experiments, N represents the number of mice from which individual samples were prepared. For FRET measurements, number of mice (N) and number of single measured cells (n) are indicated below the graphs. Statistical analysis was performed using GraphPad software. Normal distribution was tested using Kolmogorov-Smirnov test with Dallal-Wilkinson-Lille approximation to calculate *p* value. Differences were tested using one-way ANOVA followed by Sidak’s or Dunett’s multiple comparison tests, Student’s t-test or Mann-Whitney U test, as appropriate, and indicated in the figure legends. Significant differences are indicated as *p < 0.05, **p < 0.01, ***p < 0.001, and ****p < 0.0001.

## Results

### Regulation of PDE2A and PDE3B in T Cell Subsets Upon TCR Engagement

**FIGURE 1:**
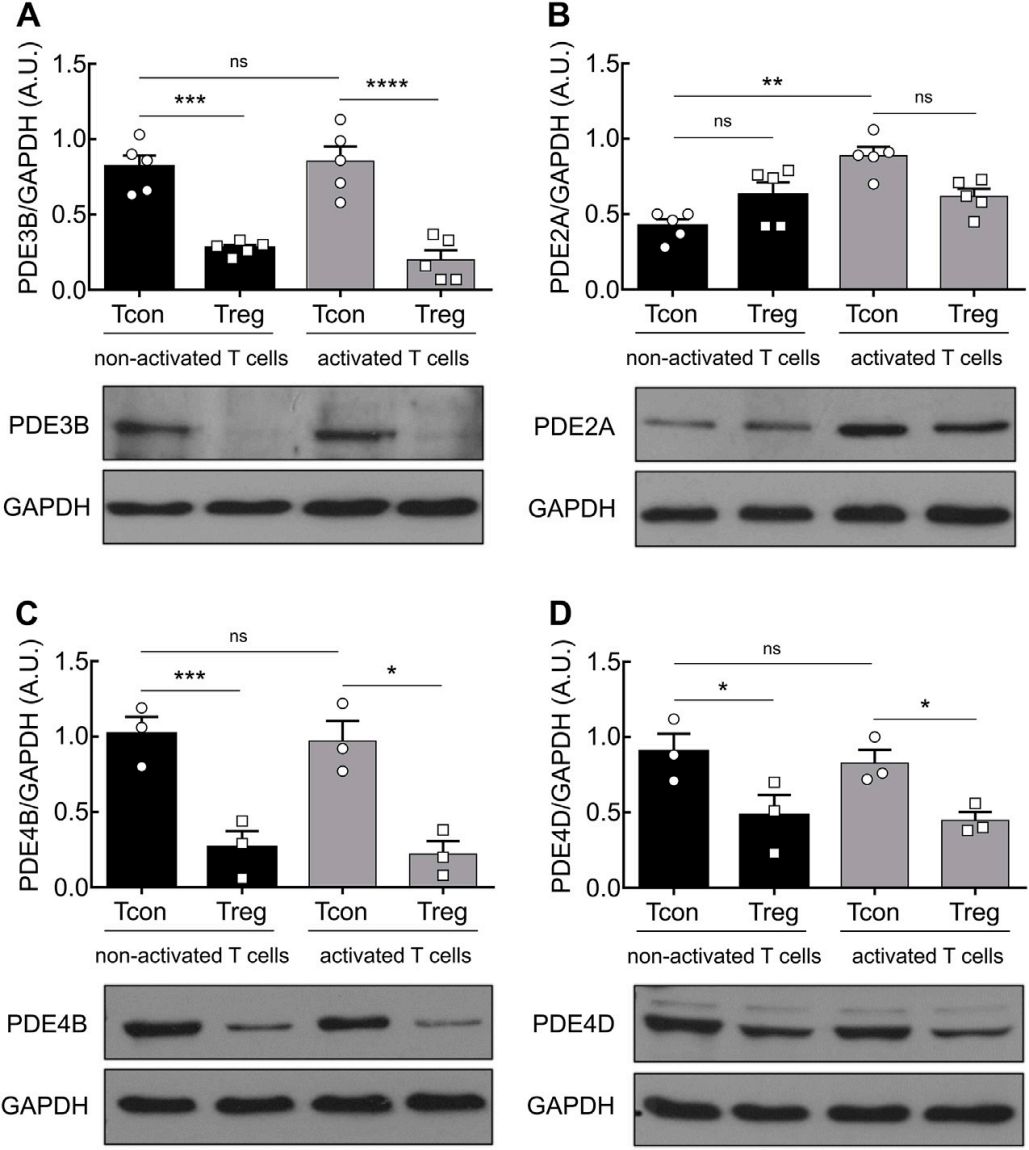
PDE2A is upregulated in Tcon upon TCR engagement. Magnetically isolated T cell subsets were cultured for 24 h in the presence or absence of anti-CD3/CD28 Dynabeads. Representative immunoblots show protein expression and quantification of **(A)** PDE3B, **(B)** PDE2A, **(C)** PDE4B, and **(D)** PDE4D. GAPDH was used as a loading control. Results in bar graphs are presented as mean ± SEM from three to five independent experiments. A.U.–arbitrary unit. *p < 0.05, **p < 0.01, ***p < 0.001, ****p < 0.0001, ns–not significant by one-way ANOVA followed by Sidak’s multiple comparison test.

Previous work that elucidated the impact of different PDEs in T cell subsets showed that the *Pde3b* gene, which encodes for the PDE isoform PDE3B, a cGMP-inhibited phosphodiesterase, is downregulated in Treg by strong Foxp3-mediated transcriptional repression (Zheng et al., 2007). However, the expression and regulation of the cGMP-activated phosphodiesterase family PDE2A in T cells have not been systematically investigated. To address the role of PDE2A and to confirm earlier findings on PDE3B in distinct T cell subsets, we analyzed the expression of both PDEs at the protein level. MACS-purified Tcon and Treg from mouse splenocytes and lymph nodes were cultured for 24 h in the absence or presence of anti-CD3/CD28 Dynabeads to induce T cell activation, followed by immunoblot analysis. We confirmed a significantly higher expression of PDE3B in both non-activated and activated Tcon as compared to Treg which barely express this PDE subfamily (Figure 1A). However, T cell activation did not have any effect on PDE3B expression. By contrast, stimulation of TCR engagement in Tcon led to a marked increase in PDE2A protein expression. Notably, this effect was detected exclusively in Tcon, whereas activation of Treg did not induce PDE2A upregulation (Figure 1B). Since the exposure of mouse thymocytes (Michie et al., 1996) and human T cells to the adenylyl cyclase activator forskolin or even TCR ligation was reported to upregulate PDE4 activity (Erdogan and Houslay, 1997) or protein levels of short PDE4B and PDE4D isoforms upon >24 h exposure (Peter et al., 2007), respectively, we tested whether TCR stimulation also changes PDE4 protein expression in mouse Tcon and Treg. Interestingly, under our experimental conditions we could not detect activation-dependent changes in PDE4B and PDE4D content (Figures 1C,D), although their expression in Tcon was constitutively higher than in Treg which is compatible with previously published data (Vang et al., 2013).

### Real-Time Monitoring of cAMP Supports Functional PDE2A Upregulation in Activated Tcon

**FIGURE 2:**
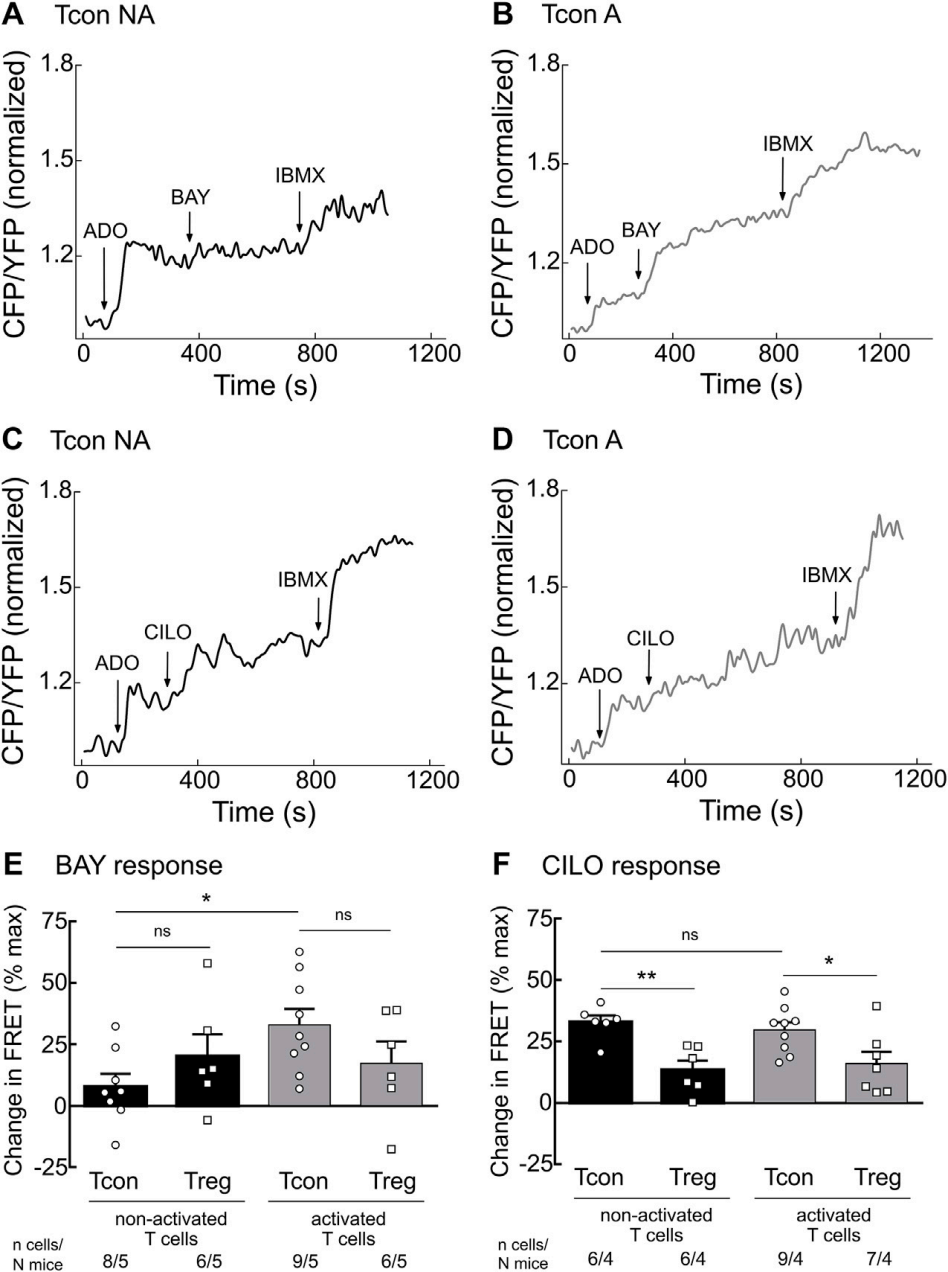
Live-cell imaging of PDE2 and PDE3 inhibitor effects in the cytosol of non-activated (NA) and activated (A) T cells. Murine Tcon and Treg isolated from CAG-Epac1-camps mice were cultured for 16–24 h in the presence or absence of anti-CD3/CD28 Dynabeads and used for FRET based live-cell recordings. Representative traces from FRET measurements for **(A)** non-activated and **(B)** activated Tcon upon PDE2A inhibition by BAY (100 nM) and **(C)** non-activated and **(D)** activated Tcon upon PDE3B inhibition by CILO (10 µM) are shown. T cells were first prestimulated with adenosine (ADO, 10 µM) to induce cAMP production. **(E,F)** Quantification of FRET responses shown in A-D, together with Treg responses, upon stimulation with **(E)** BAY or **(F)** CILO of individual cells measured are presented as percent of maximal FRET biosensor response generated by IBMX (100 µM). Decrease in cAMP level is shown as a negative value. Results are depicted as mean ± SEM. Cell number (*n*) and total number of mice (*N*) per group are indicated below the bars. *p < 0.05, **p < 0.01, ns–not significant by one-way ANOVA followed by Sidak’s multiple comparison test.

To further assess the cAMP hydrolyzing activity of PDE2A and PDE3B, we established real-time live-cell imaging of cAMP in T cells using the highly sensitive cytosolic FRET biosensor Epac1-camps (Supplementary Figure S2) (Börner et al., 2011; Sprenger et al., 2012). We isolated T cell subsets from splenocytes of transgenic mice ubiquitously expressing this biosensor (Calebiro et al., 2009) and cultured them overnight to analyze both activated and non-activated T cells. During FRET experiments, T cells were firstly stimulated with adenosine (ADO) to evoke intracellular cAMP production, followed by inhibition of specific PDEs using the selective PDE2A inhibitor BAY 60–7550 (BAY) or the selective PDE3 inhibitor cilostamide (CILO). The maximal cAMP response of the biosensor was subsequently induced by the non-selective PDE inhibitor 3-isobutyl-1-methylxanthine (IBMX). PDE2A inhibition led to significantly stronger cAMP response in activated as compared to non-activated Tcon (Figures 2A,B,E). Again, this effect was not observed for Treg (Figure 2E and Supplementary Figure S3), compatible with exclusive upregulation of PDE2A upon Tcon activation (Figure 1B). FRET responses to the PDE3 inhibitor CILO were higher in Tcon compared to Treg (Figures 2C,D,F and Supplementary Figure S3) as expected from the expression pattern of PDE3B (Figure 1A). However, no significant differences in PDE3 responses were observed in naive T cells compared to activated T cells in both Tcon and Treg (Figure 2F). Both BAY (*in vitro* IC_50_ of ∼4 nM) and CILO (*in vitro* IC_50_ of ∼70 nM) are selective for PDE2 and PDE3 (>100-fold selectivity vs other PDEs), respectively, in the concentration used (Sudo et al., 2000; Masood et al., 2009; Perera et al., 2015). To confirm their specificity, we have repeated these experiments using another pair of selective inhibitors PF-05180999 for PDE2A (Helal et al., 2018) and cilostazol for PDE3 (Schrör, 2002), which led to comparable results (Supplementary Figure S4). Thus, consistent with upregulation of PDE2A upon Tcon activation, FRET measurements also revealed higher functional activity of PDE2A in activated Tcon.

### Real-Time Measurements of cGMP/cAMP Cross-Talk Upon T Cell Activation

**FIGURE 3:**
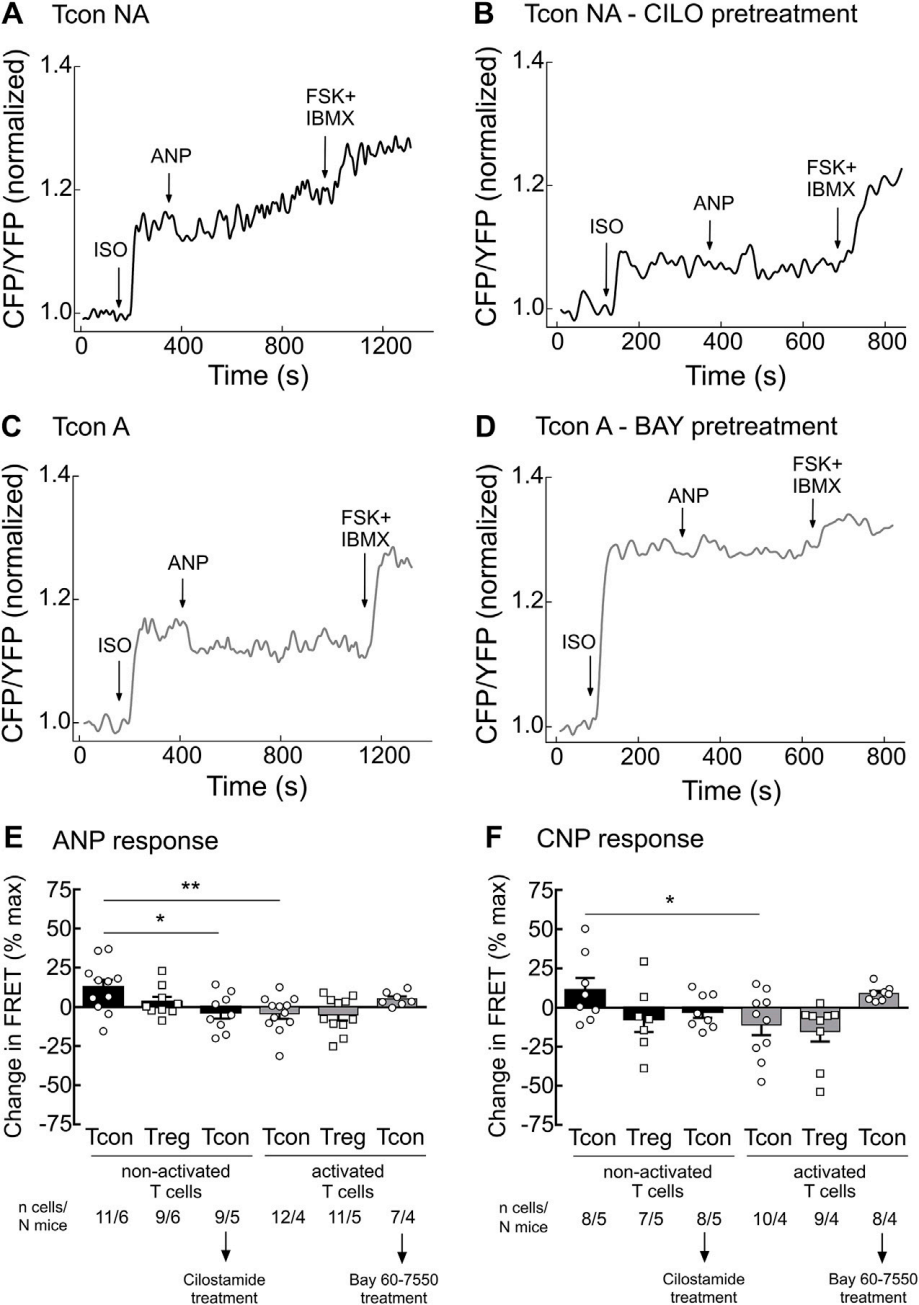
Tcon activation changes cGMP/cAMP cross-talk in response to NP. Primary Tcon and Treg were MACS-purified and cultured for 16–24 h in the presence or absence of anti-CD3/CD28 Dynabeads following FRET measurements. Representative FRET traces from Tcon expressing Epac1-camps sensor in **(A)** non-activated (NA), **(B)** non-activated T cells after CILO pretreatment (10 µM), **(C)** activated, and **(D)** activated T cells after BAY pretreatment (100 nM). T cells were initially stimulated with the non-selective β-AR agonist isoprenaline (ISO, 1 µM), to induce cAMP production. Change in FRET represents a cAMP response to NPs (200 nM ANP or 300 nM CNP) of individual cells as a percent of maximal FRET biosensor response induced by Forskolin (FSK, 10 µM) and IBMX (100 µM). Decrease in cAMP level is depicted as a negative value. **(E,F)** Quantification of FRET responses shown in **(A–D)**, together with data obtained from Treg measurements are depicted in this bar graph. Results are presented as mean ± SEM. Cell number and total number of mice measured per group are indicated below the bars. *p < 0.05; **p < 0.01; ns–not significant by one-way ANOVA followed by Sidak’s multiple comparison test.

Since the activity of PDE2A and PDE3 is modulated by cGMP, we next studied how stimulation of cGMP production in T cells affects cAMP responses. To understand this cGMP/cAMP cross-talk which is best studied in other cells types upon co-stimulation of β-AR and NP receptors (Götz et al., 2014; Perera et al., 2015), we first stimulated T cells with the unselective β-AR agonist isoproterenol (ISO) to increase cAMP production. Next, we applied saturating concentrations of the natriuretic peptides ANP or CNP on top of ISO to increase cGMP generation and explore how it modulates intracellular cAMP levels in T cells. To fully saturate the FRET sensor at the end of the experiment, we applied the adenylyl cyclase activator forskolin (FSK) together with IBMX. Real-time measurements revealed that both ANP and CNP augmented ISO-stimulated cAMP responses in non-activated Tcon (Figures 3A,E,F). This was abolished by pretreatment of T cells with CILO, suggesting that cGMP inhibits the PDE3-mediated cAMP degradation (Figures 3B,E,F). In sharp contrast, activated Tcon responded to ANP and CNP with a decrease in cAMP levels, and this effect was sensitive to the PDE2A inhibitor BAY (Figure 3C,D–F, Supplementary Figure S3). This suggests that cGMP stimulates PDE2A activity, which dominates during T cell activation, due to the induction of PDE2A expression. However, no significant difference could be observed when looking at ANP or CNP effects in Treg, with or without TCR activation (Figures 3E,F), confirming that the modulation of cGMP/cAMP cross-talk depends on the T cell activation state and PDE2A induction exclusively in Tcon.

### Effects of Natriuretic Peptide/cGMP Signaling on T Cell Activation

**FIGURE 4:**
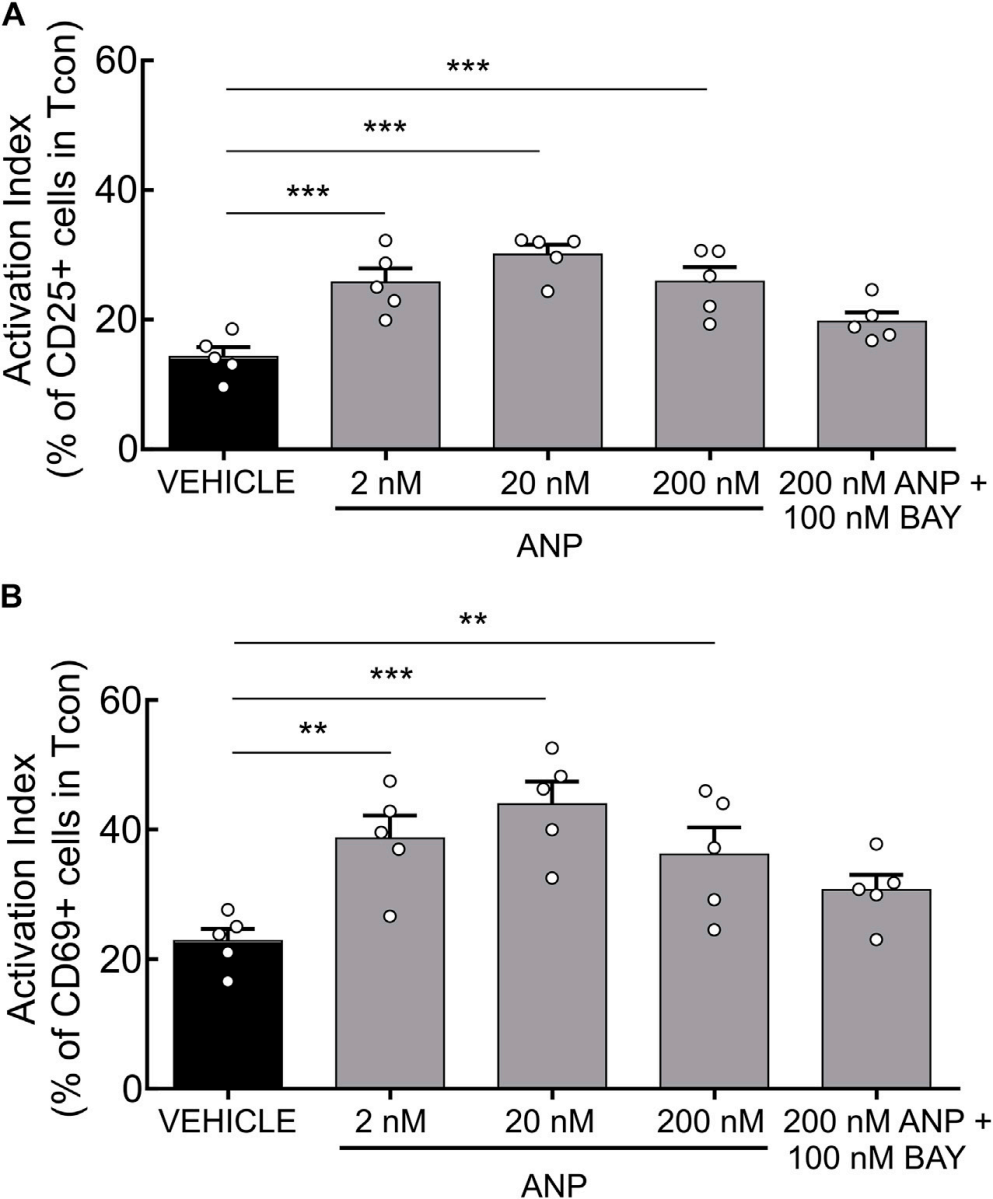
ANP stimulation enhances T cell activation in CD4^+^ T cells. MACS-purified Tcon were stimulated for 6 h in presence of anti-CD3/CD28 and ANP. Surface expression of CD25 and CD69 was assessed by flow cytometry. Results are presented as an activation index calculated as the difference of **(A)** CD25 and **(B)** CD69 expression between anti-CD3/CD28 stimulated and unstimulated CD4^+^ T cells**.** Bar graphs show quantified values of five individual animals per group. The significance of data was confirmed by repeated-measures one-way ANOVA followed by Dunett’s multiple comparison test; **p < 0.01, ***p < 0.001.

Finally, we set out to test the functional relevance of our findings and investigated the effects of ANP on T cell activation. Previously, it has been shown that increased cAMP levels inhibit TCR-induced T cell activation (Ruppelt et al., 2007; Brudvik and Taskén, 2012). Since PDE2A as a cGMP-activated PDE is upregulated during Tcon activation, we hypothesized that a rise of intracellular cGMP induced by ANP, *via* its receptor GC-A, could lower intracellular cAMP levels via PDE2A activation, further promoting T cell activation. After short-term engagement of the TCR expression of the early activation markers CD25 and CD69 on purified Tcon was significantly higher upon ANP treatment (Figures 4A,B) compared to vehicle treated cells. Co-treatment with BAY abolished the effect of ANP, confirming that GC-A/cGMP signaling acts via PDE2A to promote T cell activation (Figures 4A,B), while BAY alone had no significant effect (Supplementary Figure S5).

### PDE2A is Upregulated in Tcon During an Experimental Mouse Model of Multiple Sclerosis

Finally, to test whether upregulation of PDE2A can also be detected in *in vivo*-activated conventional T cells, we analyzed *Pde2a* mRNA expression in FACS-purified Tcon that were isolated from draining lymph nodes and spleen from control mice or mice at the peak of EAE disease severity. Indeed, in this model of multiple sclerosis we could detect a 2-fold induction of *Pde2a* (Supplementary Figure S6) in Tcon, suggesting that this mechanism might play a role in T cell activation after autoantigen immunization.

## Discussion

Degradation of cyclic nucleotides by PDEs makes these specialized enzymes interesting targets for therapeutic intervention. Since cAMP regulates pro- and anti-inflammatory activities of immune cells, drugs that elevate intracellular cAMP levels, including some PDE inhibitors, have been shown to be effective in suppressing inflammatory and autoimmune responses (Raker et al., 2016).

Earlier research of individual PDE families had focused on investigating the predominant role of PDE3, PDE4, PDE7, and PDE8 in controlling cAMP degradation in the immune system (Torphy, 1998). Pharmacological manipulation of PDE3 in mouse CD4^+^ T cells led to the enrichment of cell population with Treg-like phenotype along with the discovery of functional ability to prevent allograft rejection *in vivo*, thereby confirming the previously postulated physiological importance of PDE3 inhibition on the gene level in Treg (Gavin et al., 2007; Feng et al., 2011). Furthermore, PDE7 induction has been reported as a crucial factor during T cell activation (Li et al., 1999). Several studies uncovered the influence of PDE8 regulation in immune cells. For instance, the specific PDE8A isoform has been proven to control activated T cell motility under physiological shear stress conditions (Basole et al., 2017). Also, a few decades ago, PDE2 and PDE4 have been identified as major contributors of overall PDE activity in murine thymocytes (Michie et al., 1996). Up to now, extensive research has been done in revealing biological relevance of PDE4 in T cells, and yet, the impact of PDE2A on cAMP levels in T cell subsets remains undefined. In this study, we could uncover the selective upregulation of PDE2A in Tcon upon anti-CD3/CD28 stimulation at the protein level, while the expression of PDE2A in Treg as well as PDE3B expression in both Tcon and Treg remained unchanged upon TCR activation (Figure 1). In line with previous findings, we confirmed lower expression of PDE3B in Treg as compared to Tcon (Figure 1), which is known to occur via the well-accepted mechanism of Foxp3-dependent transcriptional repression of the *Pde3b* gene (Gavin et al., 2007). Interestingly, we could also confirm PDE2A upregulation in Tcon at the mRNA level in a more relevant *in vivo* T cell stimulation context during EAE (see Supplementary Figure S6), indicating that PDE2A might be important for T cell activation in inflammatory or autoimmune diseases.

To explore the real-time dynamics of cAMP changes in response to PDE2A or PDE3 inhibition in the cytosol of T cells we have used primary cells isolated from transgenic mice ubiquitously expressing Epac1-camps biosensor (Calebiro et al., 2009). In line with the modulation of protein expression we could show that the response to the PDE2A inhibitor BAY was more pronounced in TCR-stimulated Tcon as compared to non-activated Tcon. Also, the cAMP responses in Treg did not differ upon anti-CD3/CD28 stimulation, whereas inhibition of PDE3, induced by CILO, resulted in increased cAMP levels in both activated and non-activated Tcon as expected (Figure 2).

**FIGURE 5:**
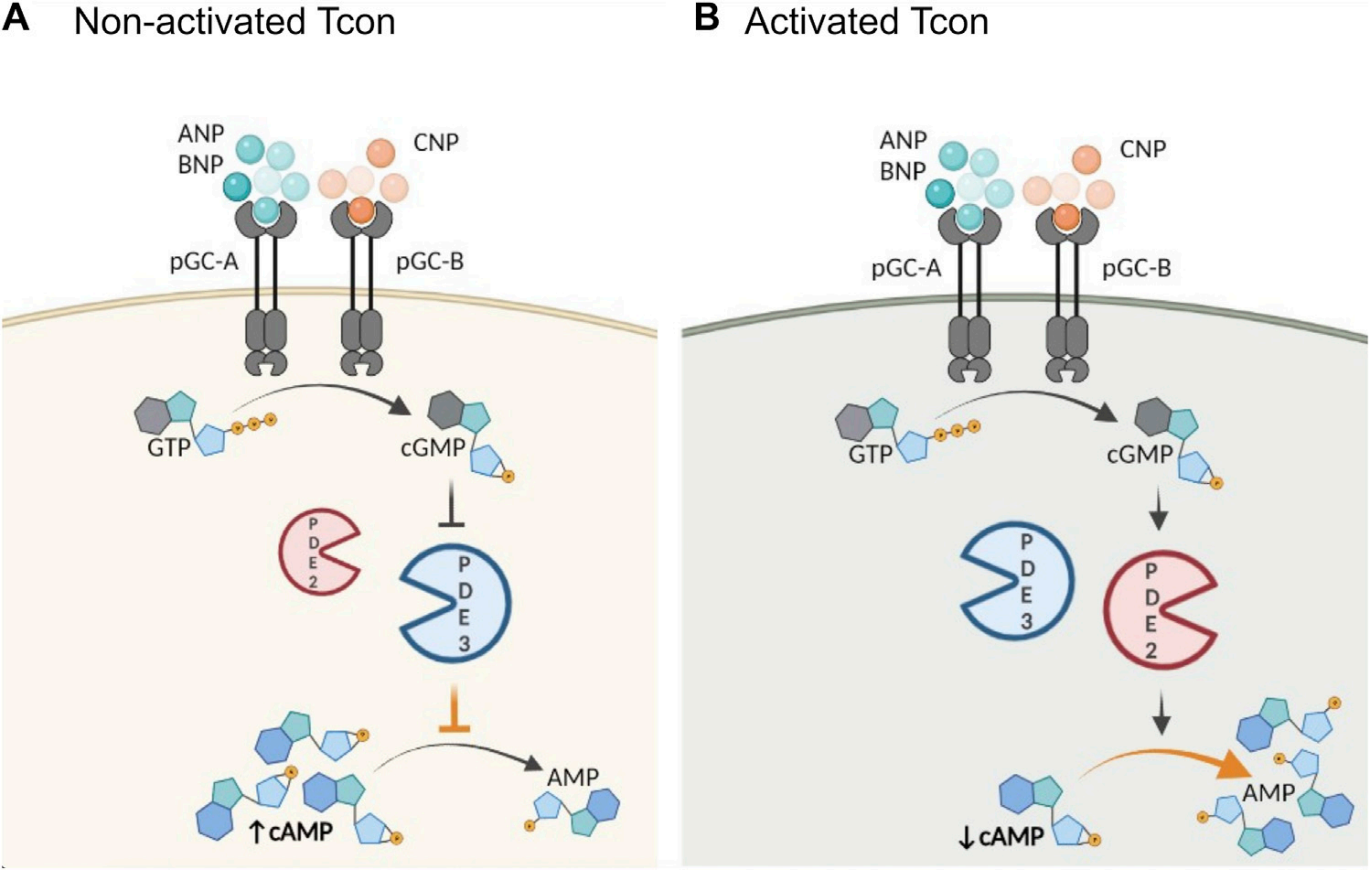
Schematic illustration of proposed role of activation and PDE2A in the cGMP/cAMP cross-talk in Tcon. cGMP generation is catalyzed by particulate GCs (pGC), membrane receptors for natriuretic peptides (NP). Central PDEs in the cross-talk are PDE2A and PDE3. **(A)** In non-activated Tcon, cGMP binds predominantly to PDE3 and acts as a competitive inhibitor of cAMP hydrolysis resulting in a positive cGMP/cAMP cross-talk loop, thereby increasing intracellular cAMP levels, while **(B)** PDE2A upregulation in activated Tcon shifts the balance to a negative cGMP/cAMP cross-talk. PDE2A is known as a dual-substrate enzyme hydrolyzing both cyclic nucleotides. Binding of cGMP to the GAF-B domain of PDE2A initiates negative cGMP/cAMP cross-talk and cAMP hydrolysis occurs at higher rate, resulting in a net decrease of intracellular cAMP. Schematic illustration was made using BioRender.

Our observations raise the question whether PDE2A upregulation and differences in cGMP/cAMP cross-talk between non-activated and activated T cell subsets have any functional implications. To uncover which specific PDE families are involved in cGMP/cAMP cross-talk in T cells, we first pretreated T cells with the β-AR agonist ISO to increase cAMP levels and then applied NPs to stimulate cGMP production. In non-activated Tcon NP/cGMP signaling further increased cAMP levels via the inhibition of PDE3, in activated T con it could rather promote cAMP hydrolysis resulting in a decrease of cAMP which was PDE2-dependent (Figures 3, 5). Therefore, our FRET data suggest that PDE3 could be the main regulator of the positive cGMP/cAMP cross-talk in non-activated T cells, whilst PDE2A upregulation in activated Tcon changes it to a negative cGMP/cAMP cross-talk in which NP/cGMP signaling reduces cAMP levels (Figure 5). Rise in intracellular cAMP typically has antagonizing effects on T cell activation, proliferation and production of pro-inflammatory cytokines (Estes et al., 1971), whereas upregulation or activation of a specific PDE family can reinforce inflammation due to increased cAMP hydrolysis. Consequently, accumulation of cyclic nucleotides by administration of PDE inhibitors serves as an attractive therapeutic strategy for inflammatory disorders. PDE inhibitors in T cells act either by limiting cytokine production by pro-inflammatory Th1 and Th17 cells or by controlling immune responses via Treg (Schepers et al., 2019). PDE4 has been identified as one of the major PDEs involved in cAMP degradation in T cells. Besides, the activity of PDE4 has been shown to be elevated in different inflammatory diseases such as asthma, psoriasis, chronic obstructive pulmonary disorders and others (Raker et al., 2016). Currently, there are three PDE4 inhibitors in clinical use to treat inflammatory disorders. PDE2 inhibitors, such as BAY, have been tested in animal models for their potential to improve neuronal plasticity and to mediate neuroprotection (Schepers et al., 2019). Specifically, in a mouse model of Alzheimer’s disease PDE2 inhibition alleviated cognitive impairment (Wang et al., 2017). The impact of PDE2A on T cell physiology, as well as the outcome of its inhibition in treating inflammation, has, to our knowledge, not been addressed yet.

Following our findings that stimulation of intracellular cGMP levels with NPs modulates the cyclic nucleotide cross-talk, we extended our research focus into exploring its functional relevance in T cell biology. NPs are peptide hormones mainly produced by the heart and thus have great importance in regulating cardiovascular physiology. However, the expression of the ANP/BNP receptor GC-A has been verified in various tissues (e.g., kidney, aortic, vascular smooth muscle, and lung), as well as in the endothelial cells expanding its involvement to diverse cellular processes (Kuhn, 2016). One older study confirmed high levels of GC-A expression and ANP/GC-A dependent cGMP production in spleen-derived naїve T cells, thereby directly linking this cardiovascular hormone with the immune system (Ma et al., 2013). Similarly, the expression of NP receptors (*Npra*, *Nprb*, and *Nprc*) have been detected in rat thymocytes as well as modulation of gene expression in response to cell activation. Moreover, ANP administration during the early phases of T lymphocyte development inhibited mitogen-activated thymocyte proliferation (Vollmar et al., 1996). Earlier studies focusing on the functional aspects of NP signaling have revealed that ANP suppressed the differentiation of Th17 cells and thereby reduced IL-17 production. This immunosuppressive effect was reversed by pretreatment of naїve CD4^+^ T cells with GC-A/cGMP-dependent protein kinase antagonists (Ma et al., 2013). Another study showed that ANP administration to asthmatic mice significantly promoted the severity of inflammatory infiltration and production of inflammatory cytokines in the lung (Ma et al., 2015), highlighting its importance in immune system regulation.

In the present study, we used an established T cell activation assay to probe the functional aspects of ANP stimulation on T cells. Since ANP increases intracellular cGMP accumulation via binding to GC-A, we hypothesized that activation of Tcon could be promoted upon ANP via PDE2-mediated hydrolysis of cAMP. It is well-known that TCR engagement provokes a transient rise in cAMP levels and that a sustained increase of intracellular cAMP results in suppression of T cell activation, proliferation and chemotaxis (Vang et al., 2001; Taskén and Stokka, 2006). In line with our hypothesis, we demonstrated that treatment of T cells with ANP during TCR engagement increases expression of the early activation markers CD25 and CD69 (Figure 4). However, PDE2 inhibition by BAY reversed this phenotype, suggesting that PDE2 upregulation during T cell activation might act as a feed-forward mechanism to support the activation by NP/cGMP signaling which decreases cAMP levels. Future studies are needed to better understand this novel mechanism in the context of T cell biology and its role in inflammation and autoimmune diseases*.*

## Data Availability

The original contributions presented in the study are included in the article/Supplementary Material, further inquiries can be directed to the corresponding author.
