# Supplementary figures and images for: Upregulation of Phosphodiesterase 2A Augments T Cell Activation by Changing cGMP/cAMP Cross-Talk
Source: Front Pharmacol. 2021 Oct 5;12:748798. doi: 10.3389/fphar.2021.748798 (PMC8523859; doi:10.3389/fphar.2021.748798)

**Full unedited blots for Figure 1.**

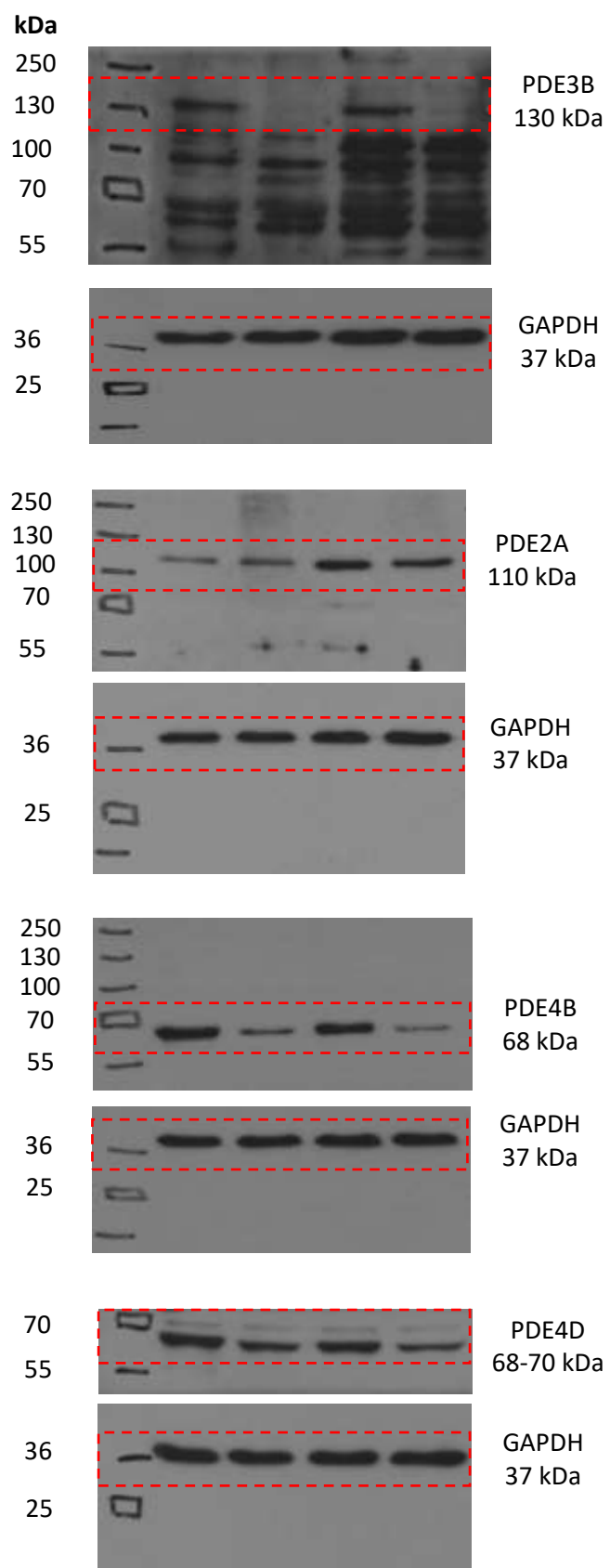

Supplement: Supplementary file 1 [file DataSheet2.PDF]
