# Supplementary material for: Upregulation of Phosphodiesterase 2A Augments T Cell Activation by Changing cGMP/cAMP Cross-Talk
Source: Front Pharmacol. 2021 Oct 5;12:748798. doi: 10.3389/fphar.2021.748798 (PMC8523859; doi:10.3389/fphar.2021.748798)
Supplement: Supplementary file 2 [file DataSheet1.pdf]

Supplementary Material

Supplementary Figures

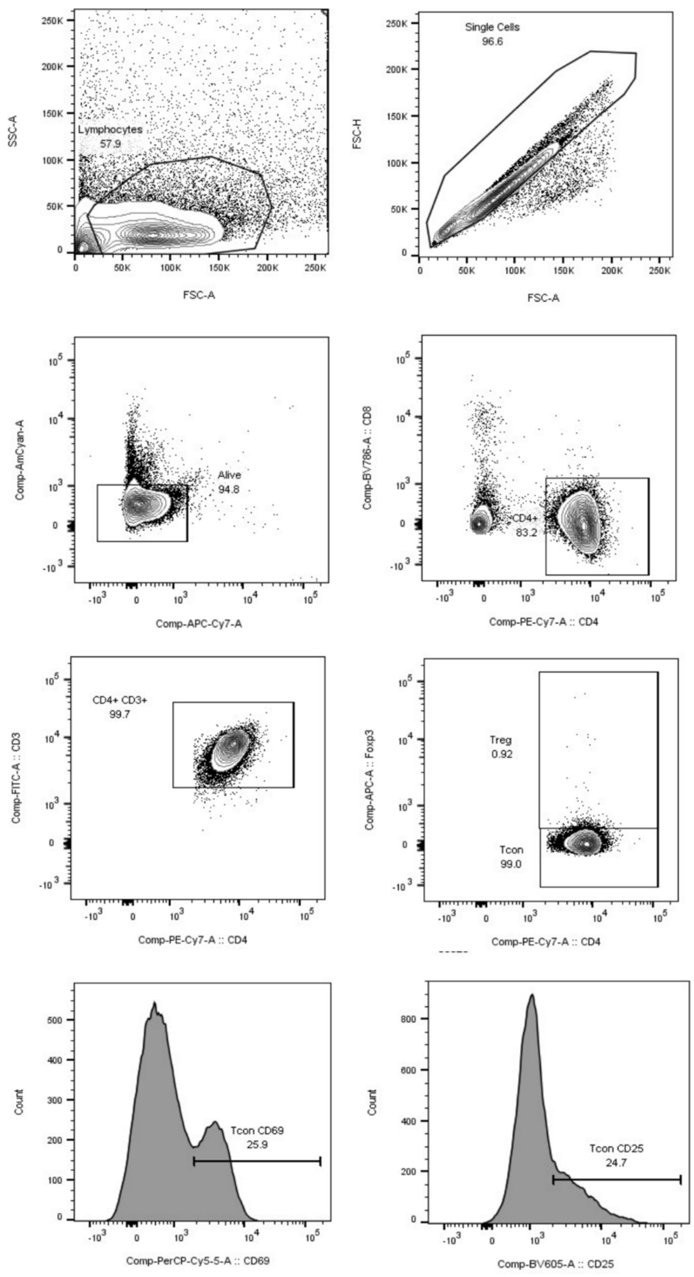

Supplementary Figure 1. Gating strategy for T cell activation assay. MACS-purified Tcon were stimulated for 6 h in presence of anti-CD3/CD28 and ANP. Surface expression of CD25 and CD69 was assessed by flow cytometry.

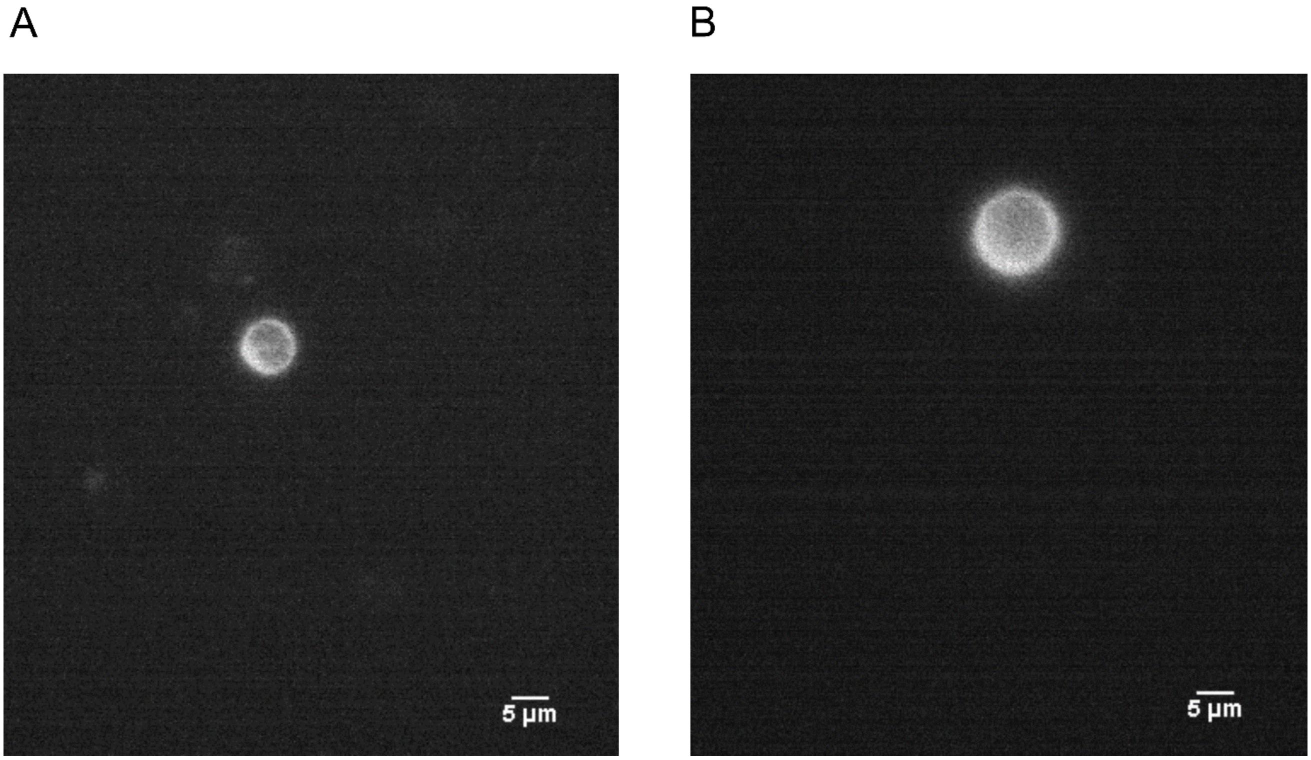

Supplementary Figure 2. Expression of Epac1-camps biosensor in T cells. Murine Tcell subsets isolated from CAG-Epac1-camps mice were cultured for 16–24 h in the presence or absence of anti-CD3/CD28 Dynabeads and used for FRET-based live-cell recordings. Representative images of cells in YFP channel showing FRET sensor expression in **(A)** non-activated and **(B)** activated T cells. Scale bar: 5 µm.

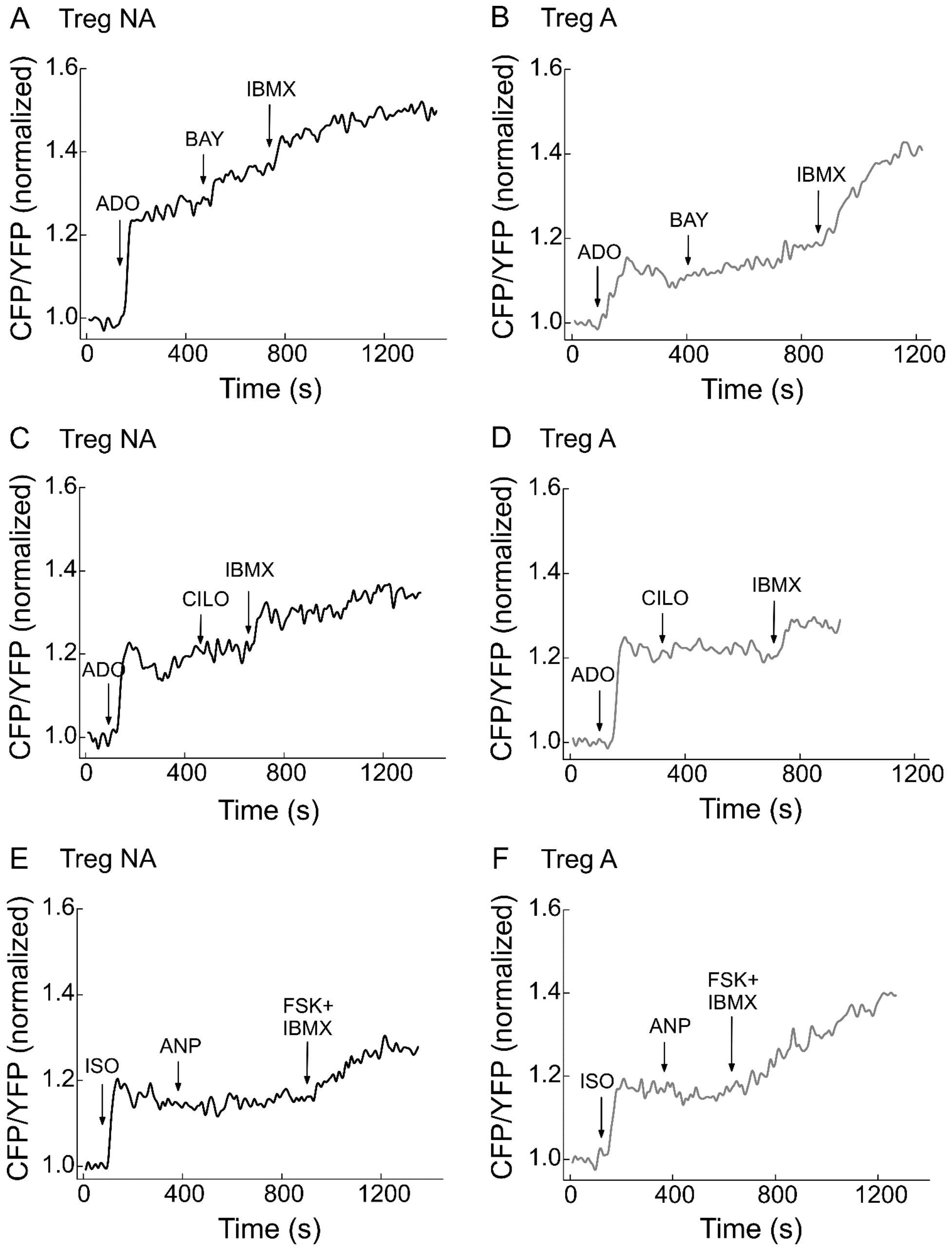

Supplementary Figure 3. Representative traces of the real-time measurements upon PDE2 and PDE3 inhibition, as well as NPs stimulation in the cytosol of non-activated (NA) and activated (A) regulatory T cells. Primary Treg were MACS purified and cultured for 16–24 h in the presence or absence of anti-CD3/CD28 Dynabeads following FRET measurements. Representative FRET traces from Treg expressing Epac1-camps sensor in **(A)** non-activated, and **(B)** activated cells after BAY treatment (100 nM), **(C)** non-activated, and **(D)** activated cells after CILO treatment (10 µM), **(E)** non-activated, and **(F)** activated cells upon stimulation with ANP (200 nM). cAMP production in T cells was induced with adenosine (ADO, 10 µM) or non-selective β-AR agonist isoprenaline (ISO, 1 µM). Maximal FRET biosensor response was induced by Forskolin (FSK, 10 µM) and/or IBMX (100 µM).

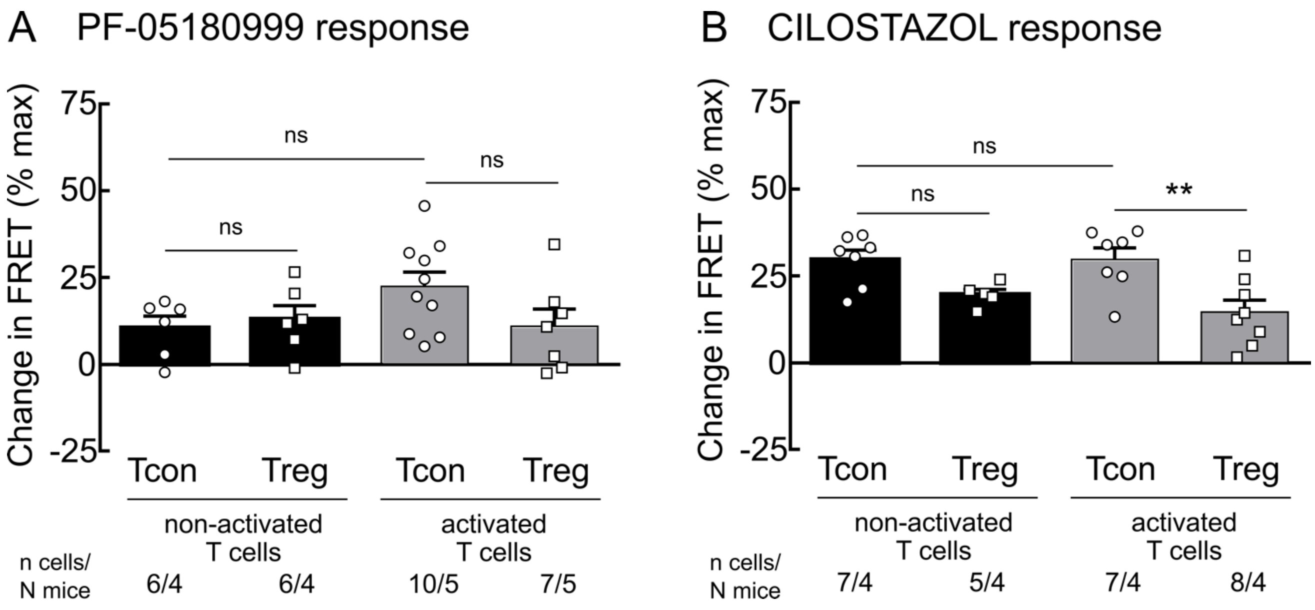

Supplementary Figure 4. Live-cell recordings of PDE2 and PDE3 inhibitor effects in the cytosol of non-activated (NA) and activated (A) T cells. Murine Tcon and Treg isolated from CAG-Epac1-camps mice were cultured for 16–24 h in the presence or absence of anti-CD3/CD28 Dynabeads and used for FRET based live-cell imaging. T cells were first prestimulated with adenosine (10 µM) to induce cAMP production. Quantification of FRET responses shown upon stimulation with **(A)** the PDE2A inhibitor PF-05180999 (100 nM) or **(B)** the PDE3 inhibitor Cilostazol (10 µM) of individual cells measured are presented as percent of maximal FRET biosensor response generated upon application of IBMX (100 µM). Decrease in cAMP level is shown as a negative value. Results are depicted as mean ± SEM. Cell number and total number of mice measurements per group are indicated below the bars. Significant differences were assessed by one-way ANOVA followed by Sidak's multiple comparison test. ns – not significant, ** $p < 0.01$.

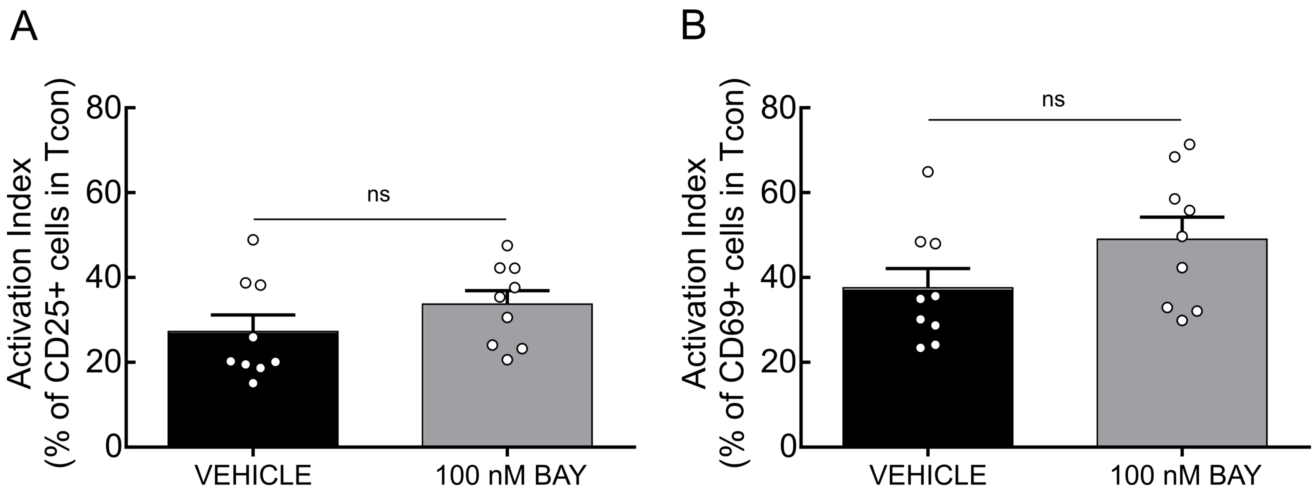

Supplementary Figure 5. Treatment with PDE2 inhibitor alone has no effect on T cell activation. MACS-purified Tcon were stimulated for 6 h in presence of anti-CD3/CD28 and BAY. Surface expression of CD25 and CD69 was assessed by flow cytometry. Results are presented as an activation index calculated as the difference of **(A)** CD25 and **(B)** CD69 expression between anti-CD3/CD28 stimulated and unstimulated CD4+ T cells. Bar graphs show quantified values of nine individual animals per group. Differences were tested by Mann-Whitney U test in A and Student′s t-test in B; ns – not significant.

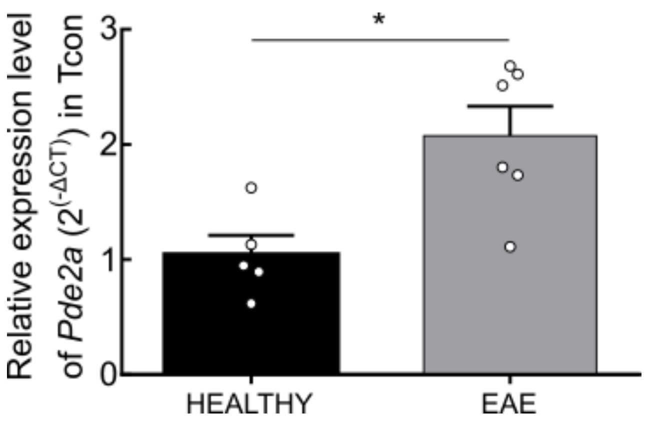

Supplementary Figure 6. Upregulation of PDE2A in Tcon of EAE-induced mice. mRNA expression of *Pde2a* was quantified by qPCR of MACS-isolated T cells from the draining lymph nodes and spleen at the peak of EAE disease course (d14/15) or from healthy control mice. *Tbp* was used as a housekeeping gene for normalization. Data are presented as relative gene expression level by using ΔCt values for quantification. * $p < 0.05$ by Student′s t-test.
